# The Australian experiment: the use of evidence based medicine for the reimbursement of surgical and diagnostic procedures (1998–2004)

**DOI:** 10.1186/1743-8462-3-3

**Published:** 2006-05-10

**Authors:** Sue P O'Malley

**Affiliations:** 1 Medical Intelligence, 13 Cudgee Street, Turramurra NSW 2074, Australia

## Abstract

**Background:**

In 1998 a formal process using the criteria of safety, effectiveness and cost-effectiveness (evidence based medicine) on the introduction and use of new medical procedures was implemented in Australia. As part of this process an expert panel, the Medical Services Advisory Committee (MSAC) was set up. This paper examines the effectiveness of this process based on the original criteria, that is, evidence based medicine.

**Method:**

The data for this analysis was sourced primarily from that made available in the public domain. The MSAC web site provided Minutes from MSAC meetings; Annual Reports; Assessment and Review reports; Progress status; and Archived material.

**Results:**

The total number of applications submitted to the MSAC has been relatively low averaging approximately only fourteen per year. Additionally, the source of applications has quickly shifted to the medical devices, equipment and diagnostic industry as being the major source of applications. An overall average time for the processing of an application is eighteen months. Negative recommendations were in most cases based on insufficient clinical evidence rather than clinical evidence that clearly demonstrated a lack of clinical effectiveness. It was rare for a recommendation, either positive or negative, to be based on cost-effectiveness.

**Conclusion:**

New medical procedures are often the result of a process of experimentation rather than formally conducted research. Affordability and the question of who should pay for the generation, collection and analysis of the clinical evidence is perhaps the most difficult to answer. This is especially the case where the new procedure is the result of a process of experimentation with an old procedure. A cost-effective way needs to be found to collect acceptable levels of evidence proving the clinical effectiveness of these new procedures, otherwise the formal processes of evaluation such as that used by the Australian MSAC since 1998 will continue to run the risk of committing Type II errors, that is, denying access to medical procedures that are beneficial and efficient.

## Introduction

Arising out of the uncertainty in decision making in any health care sector decision makers are faced with the dilemma of determining which has the greater risk, making available medical procedures that are ineffective or even harmful (Type I error) or, denying access to medical procedures that are beneficial and efficient (Type II error). Owing to the long shadow of thalidomide, there may be an over-emphasis by decision makers on the avoidance of a Type I error. Additionally, the growing availability of new technology and the resultant cost blow-outs may also have biased decision makers against making a Type I error. The combined effect may result in an unacceptable level of denying access to medical procedures that are beneficial and efficient (Type II errors). This potential dilemma can be shown diagrammatically as in Table 1.

**Table 1:** Dilemma associated with Type I and Type II Errors

	**Procedure is Beneficial**	**Procedure is Harmful**
**New procedure approved**	Correct Decision	Type 1 Error: Allowing a harmful procedure. Victims are identifiable and traceable. Error is self-correcting
**New procedure disapproved**	Type 2 Error: Disallowing a beneficial procedure. Victims are not identifiable. Error is not self-correcting	Correct Decision

In April 1998 the then Federal Minister for Health and Family Services announced that Australia had become the first nation in the world to formally adopt evidence-based medicine (EBM) as a key feature of its health system with all new medical procedures being independently evaluated by an expert panel before being admitted to the Medicare Benefits Schedule.

*"This new vetting procedure will make quality a central feature of the health system by ensuring that only medical procedures and new technologies which were safe, cost-effective and of real benefit to patients were funded through Medicare. We will no longer find ourselves in the untenable position of new procedures being used in Australia simply on the basis of anecdotal evidence or because of aggressive marketing. The introduction of evidence based medicine and the committee means that the gap between research knowledge and clinical practice will narrow, and patients will benefit earlier from the most advanced procedures drawing on the best scientific and medical evidence."*[1]

In Australia, for a medical procedure to attract funding covering the fee for the medical practitioner paid in the case of privately insured patients (43% of the population [2]), the procedure must have a Medicare Benefits Schedule (MBS) Item Number. Although this Item Number only directly determines the basic scheduled fee payable to the medical practitioner, indirectly it determines the payment of all other costs associated with the procedure (theatre, bed-days, equipment and prosthesis) since these payments are contingent on the procedure having this MBS Item Number.

Evaluation of evidence accompanying applications from the medical profession for the listing of new medical services on the Australian Medicare Benefits Schedule (MBS) is not new. The assessment of evidence has always been an integral part of the listing process of medical technologies and services on the MBS via a mix of specialist consultative and advisory bodies. The creation of the MSAC was seen as a way of formalising and strengthening this process, especially in terms of the cost-effectiveness criterion.

The guidelines for this new system of applications to the Medical Services Advisory Committee (MSAC) were loosely based on the Australian Guidelines for submissions to the Pharmaceutical Benefits Advisory Committee (PBAC) for the evaluation of all new pharmaceutical seeking listing on the Australian Pharmaceutical Benefits Schedule (PBS) that was made mandatory in Australia in January 1993 [3]. A listing on this schedule results in the pharmaceutical being subsidised by the Australian Government.

Although there are many similarities between the process used for pharmaceuticals and the new process for medical procedures, there are a number of important differences.

Submissions to the PBAC for PBS listing of a pharmaceutical include the evaluation of all the evidence by the company making the submission (usually the manufacturer or distributor). This evidence is reviewed by the Pharmaceutical Benefits Branch (PBB) and the process is totally confidential until the outcome of the submission is made public (a one page summary referred to as the Public Summary Document). In contrast, applications to the MSAC are evaluated by contractors employed by the Medicare Benefits Branch (MBB). Additionally, this process has a reasonably high level of transparency with publication on a Web site of the receipt of the application, its progress, and a detailed written report of the outcome.

Approximately one hundred submissions are received by the Pharmaceutical Benefits Branch (PBB) each year. On average, approximately nineteen applications or references are received by the Medicare Benefits Branch (MBB) for the listing of procedures.

The safety and efficacy of a pharmaceutical must already have been established by the Australian Therapeutic Good Administration (TGA) leaving only the effectiveness and cost-effectiveness to be established in the submission to the PBAC. An application to the MSAC evaluates safety, efficacy, effectiveness and cost-effectiveness of the procedure all in the one evaluation.

The time between the lodgement of the submission and the listing of the pharmaceutical on the PBS, assuming a positive outcome, is nine months [4]. A period of twenty four months between the lodgement of an application to the MSAC and the listing of the procedure on the MBS is considered to be standard.

Pharmaceuticals listed on the PBS are subsidised by the Australian Government for the whole population regardless of private health insurance status. The listing of a procedure on the MBS result in subsidy only to those covered by private health insurance, approximately 43% of the population as at December 2004 [5].

The establishment of the Medical Services Advisory Committee (MSAC) by the Australian Federal Government had three key objectives [1].

• Only medical procedures and new technologies which were safe, cost-effective and of real benefit to patients would be funded through Medicare.

• There would be a more rigorous assessment by the MSAC to ensure that the medical procedure and new technology was safe, cost effective and of real benefit to the patient.

• The gap between research knowledge and clinical practice would narrow, and patients would benefit earlier from the most advanced procedures drawing on the best scientific and medical evidence.

After more than six years of operation, it may now be timely to review how effective the MSAC has been, both in terms of screening the introduction of new procedures as well as a possible hurdle to the introduction of new technology supplied by the medical devices and equipment industry. This review of the achievement of the original objective of the MSAC is based on:

1. Usage Rate of the MSAC process and source of applications

2. Time taken for processing MSAC applications and references

3. Recommendations and outcomes of MSAC applications and references

## Method

### Data sources

The data for this analysis was sourced primarily from that made available in the public domain. The MSAC wed site provided minutes from MSAC meetings, annual reports, assessment and review reports, progress status and archived material.

In addition to this primary source, especially in the case where contradiction arose, verbal clarification was sought from various officers in the Health Technology Section and the applicants of individual applications.

### Calculation of duration of assessment

In Tables 2a and 2b, the calculation of the duration of assessment for applications was the time between acknowledgment of receipt of the application at a MSAC meeting and the date of sign-off by the Minister for Health and Ageing. Applications submitted before December 2004 that had not been signed off by the Minister have a duration shown as a number of months with a plus sign representing the duration between acknowledgment of receipt at a meeting of the MSAC and December 2004. These estimates are considered to be conservative since the application could have been received up to three months before being acknowledged at a meeting. For example, Application 1042 was acknowledged at the May MSAC meeting but was actually received 1^st ^March 2001.

**Table 2a:** Application to the MSAC and Duration of Assessment

**App.**	**Description**	**First MSAC Meeting**	**MSAC Meeting – Endorsed**	**Minister Sign Off**	**Duration in Months**	**MSAC Outcome**
1001	Advanced breast biopsy instrumentation	May'98		May'99	12	Negative
1002	Oto-acoustic emission audiometry	May'98		Aug'99	15	Positive
1003	OctreoScan¨ scintigraphy for gastro-entero-pancreatic neuroendocrine tumours	May'98		Aug'99	15	Positive
1004	Transmyocardial laser revascularization	May'98	Aug'99	Sept'99	16	Negative
1005	Visual electrodiagnosis	May'98	May'01	Jun'01	25	Partial
1006	Endoluminal grafting for abdominal aortic aneurysm	May'98		May'99	12	Partial
1007	Saline infusion sonohysterography	May'98		May'99	12	Positive
1008	Photodynamic therapy for skin and mucosal cancer	May'98		May'99	12	Negative
1009	Sacral nerve stimulation for urinary incontinence	May'98		Mar'00	22	Negative
1010	Intravascular extraction of chronically implanted permanent transvenous pacing leads	Sept'98	Aug'99	Sept'99	12	Positive
1011	Lung volume reduction surgery – for advanced emphysema	Sept'98	Feb'01	Apr'01	19	Negative
1012	Vertebral axial decompression therapy for chronic back pain	Feb'99	May'01	Jun'01	28	Negative
1013	Treatment of diseases of the inner ear using the Round Window Microcath System	Aug'99			-	Ineligible
1014	TransUrethral Needle Ablation for benign prostatic hyperplasia	May'99	Nov'01/Mar'02	May'02	36	Interim
1015	Directional, vacuum-assisted breast biopsy	May'98	Aug'99	Sept'99	16	Interim
1016	Samarium153-lexidronam for bone pain due to skeletal metastases	May'98		Aug'99	15	Positive
1017	Chelation therapy – for cardiovascular disease	Nov'98			-	Ineligible
1018–1020	Hyperbaric oxygen treatment	May'99		Feb'01	21	Partial
1022	Commercial-in-Confidence Application	May'99			-	Ineligible
1023	Placement of artificial bowel sphincters in the management of faecal incontinence	May'99	Nov'99	Nov'99	12	Negative
1024	Total ear reconstruction	May'99		Mar'00	10	Positive
1025	Provision of Positron Emission Tomography (PET) services – deferred. See Reference 02	Aug'99		Aug'00	12	Ref 02
1026	Near patient cholesterol testing using the Cholestech LDX	Aug'99	Aug'01	Sept'01	25	Interim
1027	Provision of Positron Emission Tomography (PET) services – deferred. See Reference 02	Aug'99			-	Ref 02
1028	Gamma knife surgery	Nov'99	Nov'00	Aug'01	21	Negative
1029	Brachytherapy for the treatment of prostate cancer	Nov'99	Nov'00	Feb'01	15	Interim
1030	Low intensity ultrasound treatment for the acceleration of bone fracture healing – Exogen\texttrademark bone growth stimulator	Feb'00	Nov'01	Feb'02	24	Negative
1031	Deep brain stimulation for symptoms of advanced Parkinson's disease	May'00	May'01	Jun'01	13	Interim
1032	Intravascular ultrasound	May'00	Mar'02	May'02	24	Negative
1033	Autogenous cartilage implantation	May'00			?	On hold
1034	Selective Internal Radiation Therapy for hepatic Metastases using SIR-Spheres®	May'00	Nov'01/Mar'02	Aug'02	27	Negative
1035	Genetic test for Fragile X syndrome	May'00	Mar'02	Aug'02	27	Positive
1036	Percutaneous transluminal coronary rotational atherectomy for lesions of the coronary arteries	Aug'00		Sept'02	25	Partial
1037	Advanced breast biopsy instrumentation (Note earlier application 1001)	Aug'00	Aug'01	Sept'01	13	Positive
1038	Conformal radiotherapy	Aug'00	Nov'01	Feb'02	18	Positive
1039	Photodynamic therapy with verteporfin for macular degeneration	Nov'00	Aug'01	Sept'01	10	Partial
1040	Anatomical biomodelling	Nov'00			-	Ineligible
1041	Intravascular Brachytherapy – Commercial-in-Confidence application	May'01	Aug'02	Oct'02	17	Interim
1042	Cardiac resynchronisation therapy (CRT)	May'01	Aug'05		55+	Positive
1043	Thyrogen\texttrademark (thyrotropin alfa) as a diagnostic agent for well-differentiated thyroid cancer	May'01	Aug'02	Oct'02	17	Negative
1044	Ostase immunoassay for the mass measurement of serum bone alkaline phosphatase	May'01	May'03	Aug'03	27	Negative
1045	Intra-articular viscosupplementation for treatment of osteoarthritis of the knee	Aug'01		Mar'03	19	Negative
1046	Interstim for sacral nerve stimulation in patients with refractory urinary incontinence	Aug'01	Jun'02		11+	Negative
1047	Endoluminal Gastroplication for the treatment of Gastro-Oesophageal Reflux Disease GORD	Aug'01		Jun'02	10	Negative
1048	Intradiscal electrothermal anuloplasty for patients with chronic low back pain due to anular disruption of contained herniated discs	Aug'01	Nov'02	Dec'02	16	Negative
1049	M-VAX TM – a treatment for patients with advanced stage III melanoma	Nov'01	Aug'02	Oct'02	11	Negative
1050	Optical Biometry	Nov'01	Mar'03/Aug'03	Jun'04	31	Positive
1051	Vacuum Assisted Closure (VAC) Therapy	Nov'01			-	Ineligible
1052	Radiofrequency ablation of liver tumours	Nov'01	May'03	Aug'03	21	Partial
1053	Placement of artificial bowel sphincters in the management of faecal incontinence	Mar'02	May'03	?	14+	Negative
1054	Hyperbaric Oxygen Therapy (Resubmission)	Mar'02	May'03/Aug'03	Aug'04	29	Interim
1055	Hysteroscopic sterilisation by tubal cannulation and placement of intrafallopian implants	Mar'02	Nov'03	Mar'05	36	Negative
1056	LeukoScan®	May'02	May'03	Aug'03	15	Partial
1057	M2A® capsule endoscopy – evaluation of obscure gastrointestinal bleeding in adult patients	Aug'02	Aug'03	Sept'03	16	Interim
1058	QuantiFERON-TB Gold		Aug'04		-	Withdrawn
1059	Endo Venous Laser Treatment for Varicose Veins	Mar'03	Nov'03	Aug'04	17	Negative
1060	Bone Mineral Densitometry – Reference 19	Mar'03	Nov'04		-	Withdrawn
1061	Implantation of Insertable Loop Recorder for Diagnosis of Recurrent Unexplained Syncope	Mar'03	Nov'03		8+	Positive
1062	A scan for Imaging Recurrence and/or metastases in patients with histologically demonstrated carcinoma of the colon or rectum	Mar'03	May'04	Aug'04	17	Negative
1063	Photodynamic Therapy for Verteporfin (Visudyne) for Subfoveal choroidal neovasculanisations (Commercial in Confidence)	Mar'03	Nov'03	Mar'05	24	Unchanged
1064	Three dimensional magnetic electroanatomical radiofrequency ablation for the treatment of cardiac arrhythmia's	Mar'03			-	Ineligible
1065	Sentinel Node Biopsy for Breast Cancer	Mar'03	Mar'05	Jul'05	28	Interim
1066	Drug (Sirolimus) Eluting Stents ("Commercial-in-Confidence") – Refer to Reference 21	Mar'03	Aug'03	Sept'03	-	Ref 21
1067	Genotypic resistance testing of antiretrovirals in HIV	Aug'03	Aug'04	Mar'05	19	Negative
1068	Prostate specific antigen (PSA) test	Mar'04	Mar'05	Jul'05	16	Unchanged
1069	Endoscopic ultrasound for the pre-operative staging of gastric and oesophageal neoplasms – Refer to Application 1072	Mar'04			21+	App 1072
1070	An innovative patent for tobacco smoking cessation	Mar'04			-	Ineligible
1071	Measurement of international normalised ratio (INR) in general practice	Mar'04	May'05	Jul'05	16	Negative
1072	Endoscopic ultrasound for staging pancreatic, gastric, oesophageal and hepato-biliary neoplasms	Mar'04			21+	Incomplete
1073	METVIX (Commercial-in-confidence) – referred to the PBAC	Mar'04				Withdrawn
1074	Freelight Lambda and Freelight Kappa	Mar'04			-	Withdrawn
1075	Endoscopic ultrasound for the diagnosis and pre-operative staging of Hepato-biliary neoplasms – Refer to Application 1072	Mar'04			21+	Incomplete
1076	Transurethral microwave thermotherapy (TUMT)	May'04			19+	Positive
1077	Sacral nerve stimulation for faecal incontinence	May'04	May'05	Jul'05	14	Positive
1078	Multifocal multichannel objective perimetry (MMOP)	May'04	Aug'04		3+	Negative
1079	Peripheral arterial tonometry with ascending aortic waveform analysis using the SphygmoCor system	May'04	Mar'06		19+	Incomplete
1080	Radi pressure wire	Aug'04	Nov'05		16+	Incomplete
1081	Uterine artery embolisation	Aug'04			16+	Incomplete
1082	SIR-Spheres® for the treatment of non-resectable liver tumours	Aug'04			11+	Interim
1083	Intac Implants	Aug'04			11+	Negative
1084	Uro Vysion	Aug'04	Nov'05		16+	Incomplete
1085	Carbon labelled urea breath test	Nov'04			13+	Incomplete
1087	B-Type Natriuretic Peptide – includes 1086	Aug'04			16+	Incomplete
1089	Brachytherapy for the treatment of prostate cancer	Nov'04	May'05	Jul'05	8	Interim
1090	Artificial intervertebral disc replacement – previously Reference 29	Mar'04	Nov'05		14+	Incomplete
1091	Minimally Invasive Robotic Assisted Radical Prostatectomy	Mar'05			9+	Incomplete
1092	Deep brain stimulation for the symptoms of Parkinson's disease	Mar'05			9+	Incomplete
1093	Endovascular Neurointerventional Procedures	Mar'05			9+	Incomplete

Source: MSAC Web Site:  – Accessed 6^th ^Jan'06 Correct as at end Dec'05

**Table 2b:** References to the MSAC and Duration of Assessment

**Ref No.**	**Description**	**First MSAC Meeting**	**MSAC Meeting – Endorsed**	**Minister Sign Off**	**Duration in Months**	**MSAC Outcome**
1	Prostate cancer screening					Unknown
2	Positron emission tomography (PET)	Aug'99				Interim
3	Assisted reproductive technology (ART)					Unknown
4	Nuchal translucency measurement in the first trimester of pregnancy for screening of trisomy 21 and other autosomal trisomies	Aug'99		Oct'02	38	Negative
5	Provision of pulmonary thromboendarectomy (PTE)	Aug'99	Feb'01	Mar'01	19	Positive
6a & 6b	Intracytoplasmic sperm injection (ICSI) using ejaculated sperm	Nov'00	Mar'05		52+	Positive
7a	Magnetic Resonance Cholangiopancreatography					Positive
7b	Magnetic resonance imaging – (b) Staging of endometrial and cervical carcinoma	Nov'00	Nov'01	Feb'02	15	Positive
8	Intra-operative transoesophageal echocardiography	Nov'00		Jun'02	19	Interim
9a(i)	Polymerase chain reaction (PCR) in the diagnosis and monitoring of chronic myeloid leukemia (CML)	Nov'00	Mar'03	Jun'04	43	Positive
9a (ii)	Polymerase chain reaction (PCR) in the diagnosis and monitoring of acute promyelocytic leukemia (APL)	Nov'00	Mar'03	Aug'03	33	Positive
9a (iii)	Polymerase chain reaction (PCR) testing in the diagnosis and monitoring of acut myeloid leukaemia (AML)	Nov'00	Aug'03	Sept'03	34	Positive
9a(iv)	Polymerase chain reaction in the diagnosis and monitoring of patients with BCR-ABL gene rearrangement in acute lymphoid leukaemia	Nov'00	Mar'04	Jun'04	43	Positive
9b	Antenatal Screening for Heritable Thrombophilia (Assessment Report – August 2002)	Nov'00	Aug'02	Oct'02	23	Negative
10 part 2 (i)	Positron Emission Tomography (PET) – additional indications	Nov'00	May'01	Jun'01	7	Positive
10 part 2 (ii)	Positron Emission Tomography (PET) – additional indications	Nov'00	May'01		6+	Interim
11a	Off-Pump Coronary Artery Bypass (OPCAB) with the aid of Tissue Stabiliser	Nov'00	Mar'02	May'02	18	Positive
11b	Minimally Invasive Direct Coronary Artery Bypass (MIDCAB) with the aid of Tissue Stabiliser	Nov'00	Mar'02	May'02	18	Positive
12a	Liquid based cytology for cervical screening	Aug'01	Aug'02	Oct'02	14	Negative
12b	Human papillomavirus testing in women with cytological prediction of low-grade abnormality	Aug'01	Aug'02	Oct'02	14	Negative
12c	Computer-assisted image analysis for cervical screening	Aug'01	May'03	Aug'03	24	Negative
12d	Human papillomavirus testing for cervical screening	Aug'01	May'03	Aug'03	24	Negative
12e	The use of human papillomavirus (HPV) testing to monitor effectiveness of treatment of high-grade intraepithelial abnormalities of the cervix	Aug'01	Mar'04		31+	Positive
13	Multifocal Multichannel Objective Perimetry	Nov'01	Nov'02	Dec'02	13	Negative
14	Laparascopic adjustable gastric banding for morbid obesity	Mar'02	Aug'03	Sept'03	18	Positive
15	Transanal Endoscopic Microsurgery	May'02	Mar'03		10	Positive
16	Positron Emission Tomography (PET)	Aug'02	Nov'03	Mar'05	31	Positive
17	Neonatal Hearing Screening	Aug'02	Mar'04	Mar'05	31	Unknown
18	Faecal Occult Blood Test	Aug'02	May'03			Partial
19	Bone Densitometry	Aug'02	Nov'04		27+	Withdrawn
20	Carotid Stenting	Mar'03	Mar'05	Jul'05	28	Partial
21	Drug (Sirolimus) eluting Stents – Report not published – Subsumed by Ref. 30	Mar'03	Aug'03			Ref 30
22	Treatment of hyperhidrosis of the axillae	Nov'03				Withdrawn
23	Injection of Botulinum Toxin for the treatment of spasticity following stroke					Withdrawn
24	Injection of Botulinum Toxin for the treatment of focal spasticity in adults					Withdrawn
25	Magnetic resonance cholangiopancreatograpy (MRCP)	Nov'03	Mar'05	Jul'05	20	Positive
26	Positron emission tomography (PET) for epilepsy	Nov'03	Nov'04	Mar'05	18	Positive
27	Vertebroplasty	Mar'04			12	Interim
28	Laparoscopic removal of malignancies	Mar'04				Withdrawn
29	Artificial intervertebral disc replacement – Application 1090	Mar'04	Nov'05		21+	App 1090
30	Drug eluting stents	Mar'04	Nov'04	Mar'05	12	Noted
31	Endometrial ablation for chronic ractory menorrhagia	Mar'04	Mar'05	Jul'05	16	Unchanged
32	Implantable Cardiac Defibrillators for Chronic Heart Failure	Nov'04			13+	Incomplete
33	Treatment of cerebral aneurysms	Nov'04			13+	Incomplete

Source: MSAC Web Site:  – Accessed 6^th^Jan'06

## Results

### Usage rate of the MSAC process

The MSAC system cannot be said to be fulfilling its criteria if the system is not utilised. Between April 1998 and the end of December 2004 ninety-one applications and thirty-three references had been made to the Medicare Benefits Branch, Department of Health and Ageing of the Australian Government for evaluation by the MSAC. In addition to assessments initiated by applications (applications), MSAC's terms of reference allow the Committee to undertake reviews (references) as referred by the Minister or the Department of Health and Ageing. The procedures covered by these application and references are shown in Tables 2a and 2b.

Although Tables 2a and 2b show a wide diversity of applications and references for new procedures requiring subsidy via the establishment of new MBS Item Numbers, the total number is low averaging only approximately nineteen per year. Compare this to the number of existing MBS Item Numbers. The November 2004 edition of the Medicare Benefits Schedule runs for 696 pages with 190 pages of listings for therapeutic procedures (with up to twenty Item Numbers per page), another 56 pages for diagnostic procedures and diagnostic imaging, and 25 pages of pathology services.

### Medical practitioners as a source of MSAC applications and 'Item Drift'

When the MSAC was established in 1998 it was assumed that the majority of applications would originate from the professional medical associations that represent the medical practitioners since the Medicare Benefits Schedule (MBS) is primarily a schedule of fees for the payment of the medical practitioner. A summary of the sources of MSAC application and references, divided into financial Years (1^st ^July to 30^th ^June), is shown in Table 3. In the June 2003 to July 2004 Financial Year, only one of the fifteen applications came from the medical profession. The remainder were sponsored by industry.

**Table 3:** Applications & References – Duration of Assessment

	**Applications**		**References**	
	**Number**	**Percentage**	**Number**	**Percentage**

1 – 12 months	20	27%	5	15%
13 – 18 months	26	35%	11	32%
19 – 24 months	15	20%	7	21%
25 – 36 months	12	16%	7	21%
36 + months	1	1%	4	12%
Total	74	100%	34	100%

Average (months)	17.8		22	
Range (months)	3 – 55		6 – 52	

Possibly, some early experiences with this new system resulted in alerting the medical practitioners that an MSAC application is a time consuming and risky process. An MSAC application results in attention being focused on the usage of the existing MBS Item Number to cover the new procedure. If the MSAC application is unsuccessful, the wording of the existing Item Number will more than likely be modified specifically to exclude the new procedure.

A further possible explanation for this low usage rate of the MSAC process by medical practitioners is a practice that may best be referred to as 'Item Drift'. This is the practice of an established procedure evolving into a new procedure over time with the medical practitioner's fee being claimed under the existing MBS Item Number. This practice is somewhat facilitated by the often broad and vague wording of the descriptors of the existing Item Numbers. A clear example of this is the low number of laparoscopic procedures listed on the MBS despite laparoscopic surgery being common practice. The additional time and skills required by the medical practitioner to perform a new procedure can be covered by simply increasing the gap payable by the patient (total fee charged minus amount covered by the MBS Item Number).

### Industry as a source of MSAC applications

Despite the original expectation that the majority of MSAC applications would originate from professional medical organisations, the source of applications has quickly moved to the medical devices and equipment industry as being the major, and virtually only, source of applications. In 2004 all but one MSAC application came from the medical devices and equipment industry, raising the question as to why this industry is taking such a leading role in a process primarily designed to facilitate fees for medical practitioners.

The answer lies with the nature of the new procedures. A close examination reveals that without exception all new MSAC applications cover procedures that include the use of new technology, that is, capital equipment, consumables, disposables, prostheses or medical devices. The payment for capital equipment, consumables and disposables is determined by a process referred to as Theatre Banding that determines the payment of theatre costs. The 'banding' of a procedure is directly linked to the MBS Item Number used by the medical practitioner. If the medical practitioner uses an existing MBS Item Number the new capital equipment, consumables or disposables is not covered. Similarly, the payment for prostheses or medical devices is covered by a listing on The Prostheses List. One of the criteria for a listing on this schedule is a relevant MBS Item Number. It can be difficult to explain how an MBS Item Number that has been in existence for over a decade can cover a procedure that includes prostheses new on the market in 2004. Up until 2004, the requirement for the procedure used to implant the prosthesis or medical device to be covered by a relevant MBS Item Number in order for the prosthesis or medical device to qualify for reimbursement, has rarely been enforced. However, with the rapidly growing volume and costs of prostheses and medical devices, private health insurers are now lobbying for this criterion to be enforced.

### Time taken for processing MSAC applications and references

According to the original press statement announcing the introduction of the MSAC, "patients will benefit earlier from the most advanced procedures drawing on the best scientific and medical evidence"[6]. The details of the processing times for each application or reference is shown in Tables 1a and 1b and summarised in Table 3. The overall average processing time was approximately 18 months for applications and 22 months for references.

The steps involved in processing an application can be briefly outlined as follows:

• Pre-lodgement meeting between Applicant and Health Technology Section.

• Application lodged with the Health Technology Section.

• Written information to Applicant that application has been received and deemed eligible.

• Project Officer from the Health Technology Section allocated.

• Chairperson of Advisory Panel selected from the MSAC at next scheduled meeting.

• Formation of Advisory Panel – letters sent out to relevant 'Craft Groups' for nominations.

• Evaluators appointed by Health Technology Section and requested to draft a protocol.

• First meeting of the Advisory Panel and Evaluators – refinement of draft protocol.

• Draft protocol sent out to Applicant for comments (14 days).

• Comments on draft protocol reviewed by Advisory Panel Chairperson and other members of panel if necessary.

• Evaluators evaluate application (three months).

• Evaluators draft Assessment Report presented to the Advisory Panel.

• Advisory Panel review draft Assessment Report – Evaluators incorporate changes.

• Reviewed draft Assessment Report sent out to Applicant for comments (one month).

• Response to the Applicant's comments to the Advisory Panel by Evaluators.

• Assessment Report (complete with recommendation by the Advisory Panel) and Applicant's comments presented to the next MSAC meeting.

• Recommendation of the MSAC sent to the Minister for Health and Ageing.

• Recommendation ratified by the Minister for Health and Ageing.

Note that it is only the Applicant and the Evaluators that have any time limits imposed.

The role of the Advisory Panel is to assist in the assessment of each application and provide expert input into the assessment process as well as ensuring that the Evaluator's assessment is clinically relevant. Although the Advisory panel is central to the process, it is also a major cause of delay owing to the time taken to form this panel. The formation and organisation of the first meeting of this panel can take in excess of six months. During this time the only progress made by the application is the briefing of the Evaluators and the resulting draft protocol from the Evaluators. For example, an application received in December did not have the first meeting of the Advisory Panel until the following August.

The processing times detailed in Tables 2a and 2b do not take into account the time between the lodgement of the application and the next MSAC meeting, a period of up to three months. However, far more importantly, once a decision has been ratified by the Minister, the application has to be processed by another committee, most often the Medicare Benefits Consultative Committee (MBCC) for the wording of the MBS descriptor and the determination of the fee for the service. This process is not commenced until the Minister has approved the MSAC recommendation. As a consequence a period of two years between the date of the lodgement of an application and the actual listing of the new procedure on the MBS as a claimable Item Number is not uncommon. This is clearly illustrated in Table 5 using a selection of the applications lodged for the first time in the second half of 2004.

**Table 4:** Sources of Applications and References by Financial Year

	**Applications**			**References**	**Total**
**Time Period **	**Industry**	**Individuals**	**Professional Medical Organisations**	**References**	**Total**

Apr'98 – Jun'00	14	10	11	0	35
Jul'00 – Jun'01	10	0	1	10	21
Jul'01 – Jun'02	6	1	1	3	11
Jul'02 – Jun'03	7	0	2	6	15
Jul'03 – Jun'04	14	0	1	7	22

Source: MSAC Annual Reports

**Table 5:** Processing times for Applications

**Application**	**Lodgement date**	**MSAC meeting endorsed**	**Likely MBS listing date**	Processing time*
1087	Jul'04	Jun'06	May'07	2 years 10 months
1091	Dec'04	Mar'06/Jun'06	Nov'06/May'07	1 year 11 months/2 years 5 months
1084	Jul'04	Nov'05	Nov'06	2 years 4 months
1080	May'04	Nov'05	Nov'06	2 years 6 months

* All these processing times assume that the Application is successful.

### Recommendations and outcomes of applications and references to the MSAC

Table 6 summarises the known and ratified (by the Minister for Health and Ageing) outcomes of all MSAC applications and references submitted up to the end of December 2004. It is difficult to quote a percentage of positive recommendations since these are divided into positive, partial positive and interim recommendations.

**Table 6:** Outcomes of Applications and References

**Outcome**	**Applications**					**References**	
	**Surgical**	**Therapeutic**	**Diagnostic**	**Number**	**Percentage**	**Number**	**Percentage**

Positive	5	2	8	15	17%	17	39%
Interim	5	2	4	11	13%	4	9%
Partial	2	3	2	7	8%	2	5%
Unchanged	0	1	1	2	2%	1	2%
Negative	10	9	7	26	30%	7	16%

Sub-Total	22	17	22	61	69%	31	70%

Ineligible	0	5	0	5	6%	0	0%
Withdrawn	0	2	2	4	5%	5	11%
Unknown	1	0	0	1	1%	3	7%
Other	2	0	3	5	6%	3	7%
Incomplete	5	0	7	12	14%	2	5%

TOTAL	30	24	34	88	100%	44	100%

Sourse: MSAC Web Site  accessed 6th Jan'06

A positive recommendation is basically a recommendation for an MBS Item Number covering the total indication applied for by the sponsor of the original application. A partial positive recommendation is a recommendation for an Item Number covering only part of the original indication applied for by the sponsor. An interim recommendation is temporary funding and is approved when the evidence is inconclusive but suggests that the procedure could be at least as safe, more effective, and more cost-effective than the existing comparable procedure. In these circumstances, the MSAC usually recommends interim funding for a period of three years to enable data collection and further evaluation of the procedure. These applications require a reapplication at the end of the three years based on the additional evidence collected during that time in order to maintain funding. For example, applications 1029 and 1031 that received interim funding in 2002 were re-submitted as applications 1089 and 1092. Application 1089 has been granted an extension of interim funding in order to allow time for the collection of data.

Tables 7a, 7b, 7c and 7d give a summary of the published findings from completed applications under the headings of safety, effectiveness, cost-effectiveness, comment and type. A simple qualitative analysis of these tables brings up some interesting points.

**Table 7a:** Applications with Positive Recommendations

**App.**	**Description**	**Safety**	**Effectiveness**	**Cost-effectiveness**	**Comment**	**Type**
1002	Oto-acoustic emission audiometry	Only risk – false negative/positive results	Significant variation of results	Not undertaken	The use of this technology appears to allow earlier identification of hearing impairment at less cost than alternative forms of testing.	Diagnostic
1003	OctreoScan¨ scintigraphy for gastro-entero-pancreatic neuroendocrine tumours	Safe	Sensitivity and specificity could not be determined	Not possible due to lack of data	GEP neuroendocrine tumours are relatively rare. Estimates of the incidence of carcinoid tumours vary between 7 and 13 cases per million population per year.	Diagnostic
1007	Saline infusion sonohysterography	Safe	Effective	Cost-effective if resulting in prevention of other service	... as a second-line diagnostic procedure for abnormal uterine bleeding, when findings from transvaginal ultrasound are inconclusive.	Diagnostic
1010	Intravascular extraction of chronically implanted permanent transvenous pacing leads	Complications uncommon	Effective	Insufficient data	It is a much longer, more difficult and skilled procedure than extraction of leads not entrapped by fibrous tissue, which is performed by simple traction without the use of surgical tools. Currently, both procedures are remunerated at the same rate.	Surgical
1016	Samarium153-lexidronam for bone pain due to skeletal metastases	As safe as alternative	Effective	Not done but less costly than alternative	Carcinoma of the prostate or breast – second line treatment	Therapeutic radiopharmaceutical
1021	Hepatitis C viral load testing	Safe	Effective	May be cost-effective if good patient selection	.... only used for patients with confirmed hepatitis C (by ELISA or PCR test) who undertake antiviral therapy. Other restrictions apply.	Diagnostic
1024	Total ear reconstruction	Complication rate high but acceptable	Only one low level evidence trial	Insufficient data	... only a small number of procedures (15 to 20) are therefore expected to be performed each year.	Surgical
1035	Genetic test for Fragile X syndrome	No adverse events reported	Specificity was consistently high	$14,000 – $28,000 per initial case found	Nucleic Acid Amplification (NAA) in those with specific clinical features of Fragile X (A) syndrome, including intellectual disabilities, and in first and second degree relatives of individuals with the Fragile X (A) mutation and Southern Blot where the results of NAA testing are inconclusive.	Diagnostic
1037*	Advanced breast biopsy instrumentation (Note earlier application 1001)	Safety data differs widely	Low level data only	Insufficient evidence	... public funding should be supported for the diagnostic use of this procedure, as long as fees are such that health system costs do not exceed those of comparators.	Diagnostic – Biopsy
1038	Conformal radiotherapy	May result in reduced toxicity	Similar efficacy to comparator	More effective and less costly	.... based on the additional costs of MLC alone, CRT appears to be both more effective and less costly than standard radiotherapy (RT) in some patients groups.	Therapeutic – radiotherapy
1042	Cardiac resynchronisation therapy (CRT)	Appears to be safe	As effective as pharmacotherapy		Patients who have moderate to severe chronic heart failure (NYHA class III or IV) despite optimised medical therapy, sinus rhythm, a left ventricular ejection fraction of less than or equal to 35% and a QRS duration greater than or equal to 120ms.	Surgical
1050	Optical Biometry	Safe	Accuracy is statistically superior to that of the commonly used AUS	May be less costly	PCI may be a less costly measurement technique than AUS or IUS and that it offers comparable results to ultrasound techniques.	Diagnostic – Biometry
1061	Implantation of Insertable Loop Recorder for Diagnosis of Recurrent Unexplained Syncope	Unpublished as yet	Unpublished as yet	Unpublished as yet	Patients with recurrent syncope who have had appropriate prior investigations.	Diagnostic
1076	Transurethral microwave thermotherapy (TUMT)	Unpublished as yet	Unpublished as yet	Unpublished as yet	Patients with moderate to severe symptoms of benign prostatic hypertrophy.	Surgical
1077	Sacral nerve stimulation for faecal incontinence	Evidence of safety	Some evidence of effectiveness	Some evidence of cost effectiveness	The total number of patients is small; there is some evidence of effectiveness and cost-effectiveness.	Surgical

Source: MSAC Reviews  . Accessed 6th Jan'06

**Table 7b:** Applications with Partial Positive Recommendations

**App.**	**Description**	**Safety**	**Effectiveness**	**Cost-effectiveness**	**Comment**	**Type**
1005	Visual electrodiagnosis	No significant risks identified	No rigorous evidence to support diagnostic accuracy	Could not be evaluated due to insufficient evidence	Funded – well-established tests: -electroretinography; pattern electroretinography; dark adaptometry; electrooculography; visual evoked responses. Not funded – insufficient evidence: – focal electroretinography; multifocal electroretinography; multifocal visual evoked potential; scotopic threshold response; intensity response function.	Diagnostic
1006	Endoluminal grafting for abdominal aortic aneurysm	Long term could not be established	Have not been established	No rigorous Australian cost comparison	The current MBS items for abdominal aortic aneurysm be restricted to open aortic repair; but endoluminal repair continue to receive public funding under alternative arrangements.	Surgical
1018–1020	Hyperbaric oxygen treatment	Some risk	In some indications	Could be cost effective in some indications	Funded: – decompression illness, gas gangrene, air or gas embolism; diabetic wounds including diabetic gangrene and diabetic foot ulcers; necrotising soft tissue infections including necrotising fasciitis and Fournier's gangrene, and the prevention and treatment of osteoradionecrosis. Not funded – insufficient evidence: – thermal burns, non-diabetic wounds and decubitus (or pressure) ulcers, necrotizing arachnidism, actinomycosis, soft tissue radionecrosis, osteomyelitis, skin graft survival, multiple sclerosis and cerebral palsy, cardiovascular conditions including acute myocardial infarctions, cerebrovascular disease, and peripheral obstructive arterial disease (POAD), soft tissue injuries including acute ankle sprains and crush injuries, facial paralysis (Bell.s palsy), cluster and migraine headaches, Legg-Calve-Perthes disease (necrosis of the femoral head, especially prevalent in children), sudden deafness and acoustic trauma, Crohn.s disease, osteoporosis, cancer, carbon monoxide poisoning, cyanide poisoning, head trauma, cerebral oedema, acquired brain injury, cognitive impairment, senile dementia, glaucoma, keratoendotheliosis, HIV infection, anaemia from exceptional blood loss, insulin- dependent diabetes mellitus, facial neuritis, arthritis, spinal injuries and non-union of fractures.	Therapeutic
1036	Percutaneous transluminal coronary rotational atherectomy for lesions of the coronary arteries	Insufficient data	Where conventional angioplasty and stenting cannot be undertaken successfully	Could not be determined	Funding: – revascularisation of complex and heavily calcified coronary artery lesions which cannot be treated by percutaneous transluminal coronary angioplasty (PTCA) alone or when previous PTCA attempts have not been successful; revascularisation of complex and heavily calcified coronary artery stenoses where coronary artery bypass graft (CABG) surgery is contra-indicated. Not funded: – revascularisation of coronary artery stenoses which can be satisfactorily treated by PTCA alone, with or without stent placement; revascularisation of coronary artery in-stent restenoses as a result of prior coronary artery intravascular interventions (since no long-term data exist and short-term data are conflicting).	Surgical
1039	Photodynamic therapy with verteporfin for macular degeneration	Relatively high and precise number of adverse events	More effective than placebo in patients with classic choriodal neovascularisation	Modeling suggests a cost per vision year gained of $6,100-$35,400	Funded only for patients with predominantly classic (>50% classic) subfoveal choroidal neovascularisation secondary to MD, a small minority of MD cases. For this sub-group of MD patients, there is some evidence that the therapy may retard the rate of visual loss in the short term.	Therapeutic
1052	Radiofrequency ablation of liver tumours	No significant differences in complications	Statistically significant benefit for RFA over PEI in one RCT	More expensive	Funded: – percutaneous treatment of non-resectable hepatocellular carcinoma not being considered for surgical resection. Not funded: – insufficient evidence – colorectal metastases (CLM); neuroendocrine liver metastases (NLM).	Therapeutic
1056	LeukoScan®	Low probability of adverse events	Diagnostic accuracy not significantly different	Incremental cost is $24,056 and $26,348	LeukoScan is safe and as effective as current methods of WBC scanning, but is more costly. Additional funding is justified for patients who do not have access to ex-vivo WBC scanning.	Diagnostic – Radiopharmaceutical

Source: MSAC Reviews  Accessed 6th Jan'06

**Table 7c:** Applications with Interim Recommendations*

**App.**	**Description**	**Safety**	**Effectiveness**	**Cost-effectiveness**	**Comment**	**Type**
1014	TransUrethral Needle Ablation for benign prostatic hyperplasia	Relatively safe	Relatively effective procedure for the short-term management	Additional clinical data is required	Limited role as an alternative treatment for symptomatic benign prostatic hyperplasia with the following restrictions: men with moderate to severe lower urinary tract symptoms that require specific treatment (ie those who would normally be recommended for TURP); not be medically suitable for TURP.	Surgical
1015	Directional, vacuum-assisted breast biopsy	Safe	Seems to be more effective	Not undertaken	Currently claimable under the MBS. The costs associated with the procedure should be investigated; and pending review of the costs, the procedure should receive interim funding at a higher remuneration than is currently available under existing items for nonpalpable breast lesions.	Diagnostic
1026	Near patient cholesterol testing using the Cholestech LDX	Safe	Improved diagnostic accuracy over conventional	Problematic due to uncertainties	The unrestricted use of near patient cholesterol testing using the Cholestech LDX is not recommended. The restricted use of near patient cholesterol testing, as an alternative to laboratory testing of lipids, should be considered in settings or circumstances where there is adequate training, accreditation and quality assurance.	Diagnostic
1029	Brachytherapy for the treatment of prostate cancer	May offer less risk	There has not been a successful randomised controlled trial	Slightly higher direct budgetary costs but may involve less indirect costs	Patients with prostate cancer at clinical stages T1, T2a or T2b, with Gleason Scores of less than or equal to 6, prostate specific antigen (PSA) of less than or equal to 10 ng/ml, a gland volume less than 40 cc and with a life expectancy of more than 10 years; and where the treatment is conducted at approved sites.	Surgical
1031	Deep brain stimulation for symptoms of advanced Parkinson's disease	limited evidence suggests	Some evidence – more long-term studies of improved methodological quality are needed.	Costs more than alternative over 5 years.	For patients where their response to medical therapy is not sustained and is accompanied by unacceptable motor fluctuations; and subject to the patients' participation in an appropriate controlled trial to obtain information on adverse events, longer-term patient outcomes and costs in the Australian setting.	Surgical
1041	Intravascular Brachytherapy – Commercial-in-Confidence application	Acceptable	Based on level II and III-3 evidence.	Estimated to be approximately $31,500 per TLR prevented.	There is insufficient evidence on the safety and effectiveness of implanting radioactive stents to support public funding for this procedure. The short- and medium-term data on the safety and effectiveness of catheter based intravascular brachytherapy for the treatment of coronary artery restenosis is sufficient to warrant interim funding for this procedure.	Surgical
1054^1^	Hyperbaric Oxygen Therapy (Resubmission)	Adverse events self-limited and resolved after termination of therapy	RCT evidence was of low methodological quality, failing to meet relevant validity criteria.	Clinical evidence was inadequate to substantiate claims that (HBOT) was cost-effective.	The clinical evidence was inadequate to substantiate claims that hyperbaric oxygen therapy (HBOT) was cost-effective in the treatment of refractory soft tissue radiation injuries or non-diabetic refractory wounds. However, MSAC recommended that, as there are no effective alternative therapies and in view of the progress of local data collections and an international trial, funding for HBOT continue.	Therapeutic
1057	M2A® capsule endoscopy for the evaluation of obscure gastrointestinal bleeding in adult patients	Infrequent and mild adverse events.	Little available data on this technology's effect on patient management and long-term clinical outcomes.	Lower total health care costs overall.	Funding should be supported for this procedure for patients with confirmed recurrent obscure gastrointestinal bleeding following previous colonoscopy and endoscopy without identifying bleeding source.	Diagnostic
1065	Sentinel Node Biopsy for Breast Cancer	Appears to be safe	Long term outcomes are uncertain	Based on cost minimisation, the cost to avoid lymphoedema. Therefore, the procedure appeared cost-effective.	Long term outcomes are uncertain. Funding for sentinel node biopsy should be provided pending the outcome of trials already in progress and should be reviewed in five years.	Diagnostic – Biopsy
1082	SIR-Spheres® for the treatment of non-resectable liver tumours				Patients with hepatic metastases secondary to colorectal cancer which are not suitable for resection or ablation. Not funded for non-resectable, non-ablatable hepatocellular carcinoma.	Therapeutic
1089^2^	Brachytherapy for the treatment of prostate cancer	Unchanged from 1029	Unchanged from 1029	Unchanged from 1029	As a result of re-assessment of further evidence – interim public funding should continue for patients with prostate cancer at clinical stages T1 and T2 with Gleason Scores of less than or equal to 6, prostate specific antigen (PSA) of less than or equal to 10 ng/ml, gland volume less than 40 cc and with life expectancy of more than 10 years.	Surgical

^1 ^Re-application for Application 1018–20

^2 ^Re-application for 1029

* As a general rule all interim funding is subject to data collection and is for a period of three years.

Source: MSAC Reviews  Accessed 6th Jan'06

**Table 7d:** Applications with Negative Recommendations

**App.**	**Description**	**Safety**	**Effectiveness**	**Cost-effectiveness**	**Type**
1001	Advanced breast biopsy instrumentation	Safe	Available evidence does not suggest	Not undertaken	Diagnostic – biopsy
1004	Transmyocardial laser revascularization	Carries risk & adverse events	Studies do not demonstrated/sustained effect not proven	Savings if effects sustained	Therapeutic
1008	Photodynamic therapy for skin and mucosal cancer	Safe	Trials too small, unproven	Not available	Therapeutic
1009	Sacral nerve stimulation for urinary incontinence	Relatively high adverse event rate	Uncertain long term	Expensive	Surgical
1011	Lung volume reduction surgery – for advanced emphysema	Differs widely	Limited data – not possible to determine long term	Insufficient information	Surgical
1012	Vertebral axial decompression therapy for chronic back pain	Detailed evidence lacking	Some/insufficient	No evidence based conclusion can be drawn	Therapeutic
1023	Placement of artificial bowel sphincters in the management of faecal incontinence	Could not be assessed due to insufficient data	Lack of rigorous studies – not demonstrated	Could not be assessed	Surgical
1028	Gamma knife surgery	Lack of data – unproven	Uncertain	Lack of safety & effectiveness data	Surgical
1030	Low intensity ultrasound treatment for the acceleration of bone fracture healing – Exogen\texttrademark bone growth stimulator	Safe for adults	Trial results contradictory	Unfavourable	Therapeutic
1032	Intravascular ultrasound	Relative safe overall	Insufficient evidence	Insufficient evidence	Diagnostic
1034	Selective Internal Radiation Therapy for hepatic Metastases using SIR-Spheres®	Limited evidence	Some but insufficient evidence	Not possible due to unreliable evidence	Therapeutic
1044	Ostase immunoassay for the mass measurement of serum bone alkaline phosphatase	No safety risk	Insufficient evidence	Not performed	Diagnostic
1045	Intra-articular viscosupplementation for treatment of osteoarthritis of the knee	May be at least as safe as alternatives	Limited evidence – as effective as alternatives	Little valid information but more expensive	Therapeutic
1046	Interstim for sacral nerve stimulation in patients with refractory urinary incontinence	Adverse event incidence relatively high	Effective but unproven for longer than 12 months	Not published	Surgical
1047	Endoluminal Gastroplication for the Treatment of Gastro- Oesophageal Reflux Disease (GORD)	Limited evidence – more data required	May be effective – Limited good quality data	Insufficient data	Surgical
1048	Intradiscal electrothermal anuloplasty for patients with chronic low back pain due to anular disruption of contained herniated discs	Low complications	Based on low level evidence	Insufficient evidence	Surgical
1049	M-VAX TM – a treatment for patients with advanced stage III melanoma	Unable to determine due to poorly reported data	Not possible to assess due to low level evidence	Insufficient data	Therapeutic
1053	Placement of artificial bowel sphincters in the management of faecal incontinence	Number of safety issues	Misleading findings	Not possible due to lack of clinical evidence	Surgical
1054	Hyperbaric Oxygen Therapy (Resubmission)	High rate adverse events	Studies flawed – uncertain benefits	Not possible	Therapeutic
1055	Hysteroscopic sterilisation by tubal cannulation and placement of intrafallopian implants	Appears to be relatively safe – short term data	Appears to be relatively effective – short term data	No evidence	Surgical
1059	Endo Venous Laser Treatment for Varicose Veins	Safe	Insufficient evidence	Insufficient evidence	Therapeutic
1062	A scan for Imaging Recurrence and/or metastases in patients with histologically demonstrated carcinoma of the colon or rectum	Safe	Insufficient evidence	Insufficient evidence	Diagnostic
1067	Genotypic resistance testing of antiretrovirals in HIV	Appears to be safe	Insufficient evidence	Insufficient evidence	Diagnostic
1071	Measurement of international normalised ratio (INR) in general practice	No excessive safety concerns	Limited data	Limited to direct costs – uncertainty of effectiveness	Diagnostic
1078	Multifocal multichannel objective perimetry (MMOP)	Safe	Insufficient evidence	Could not be determined	Diagnostic
1083	Intac Implants		Evidence pertaining to this procedure is immature and small in volume.	Lack of published comparative clinical studies does not allow for any cost effectiveness analysis	Surgical

Source: MSAC Reviews  Accessed 6th Jan'06

As would be expected, any procedure that had a serious safety concern was not recommended. Applications that related to a procedure likely to be carried out on a small number of patients were more likely to be given a positive recommendation. Although often applications with positive or partial positive recommendations were based on 'solid' clinical evidence of effectiveness, this was not always the case, especially in the case of interim funding. Importantly, negative recommendations were in most cases based on insufficient clinical evidence rather than clinical evidence that clearly demonstrated a lack of clinical effectiveness. It was rare for a recommendation, either positive or negative, to be based on cost-effectiveness since less than 10% of the literature search carried out by the evaluators resulted in finding any acceptable papers covering this criterion. Although, logically, this was to be expected for applications with negative recommendations due to insufficient clinical evidence, what was unexpected was that it appeared to be equally true for those applications with positive recommendations. Diagnostic procedures have a much higher total positive, partial positive or interim recommendation rate compared to surgical or therapeutic procedures. Therapeutical procedures were far more likely to be ineligible compared to surgical or diagnostic procedures.

The number of negative recommendations based on insufficient clinical evidence raises the important issue of what level of clinical evidence is sufficient. With pharmaceuticals the gold standard is the Phase Three, double blind, randomised, head-to-head clinical trial with statistically significant outcomes. In 1995, the National Health and Medical Research Council (NHMRC) prescribed a schedule of levels of evidence by which the efficacy of treatments could be assessed [7]. The 1999 revised version of these levels of evidence is shown as Table 8.

**Table 8:** NHMRC Levels of Evidence

**Level**	**Study design**
I	Evidence obtained from a systematic review of all relevant randomised controlled trials.
II	Evidence obtained from at least one properly designed randomised controlled trial.
III-1	Evidence obtained from well-designed pseudo-randomised controlled trials (alternate allocation or some other method.
III-2	Evidence obtained from comparative studies (including systematic reviews of such studies) with concurrent controls and allocation not randomised, cohort studies, case-control studies, or interrupted time series with a control group.
III-3	Evidence obtained from comparative studies with historical control, two or more single-arm studies, or interrupted time series without a parallel control group.
IV	Evidence obtained from case series, either post-test or pre-test/post-test.

Source: MSAC Reviews  Accessed 6th Jan'06

However, the generation of evidence for a medical procedure has to take into account a number of factors that do not apply to a pharmaceutical. These include:

A new medical procedure often results from a process of experimentation and variation of an existing established procedure. Small incremental changes, over time, can result in a new procedure substantially different from the original procedure. However, it is unclear at what point the procedure became a 'new' or different procedure.

Clearly, a pharmaceutical company has a financial incentive in investing in running a clinical trial on their product. However, in the case of a procedure the identification of who should carry out the clinical trial is far less clear. Individual medical practitioners or their association do not have the financial resources or the incentive.

In the case of a procedure that involves an implantable prosthesis or an item of capital equipment, the manufacturer or distributor may have an incentive to carry out the clinical trial. However, even if a medical device company does go to the expense of running a clinical trial resulting in a successful application to the MSAC, the resulting new MBS Item Number cannot be restricted to the use of that company's product. Any similar product from a competitor can be used in the procedure. This reduces the possible competitive advantage and thus incentive to carry out the clinical trial.

The best timing of a clinical trial for a procedure can be difficult to determine. If the trial is carried out too early the outcome may not be optimal due to the lack of the medical practitioner's experience or practice in performing the new procedure. Conversely, a delay in running the clinical trial results in a reduction in the 'window of time' on the return of the financial investment in the clinical trial and the new technology.

In the case of a procedure that involves new technology, there is the additional timing problem created by the on-going development and refinement of the prosthesis or capital equipment. Unlike a pharmaceutical that enters the market as a finished product, prosthesis and capital equipment continue to evolve once in the market based on feedback from the medical practitioners and patients, resulting in newer and better versions. A clinical trial based on the first version will often generate less than optimal results. However, a delay in carrying out the clinical trial and the resulting delay in funding reduces the financial viability of the product.

Clinical trials with statistically significant outcomes are expensive to conduct. Unlike pharmaceuticals, where the potential market is often measured in tens of millions of dollars per annum, the market for prostheses and capital equipment is far smaller. This limits the affordability of clinical trial covering procedures.

Clearly, there are a number of challenges involved in the generation of high level clinical evidence for medical procedures. At the same time, a public funding body such as the Department of Health and Ageing of the Australian Government cannot risk giving approval for a medical procedure that has not been proven to be safe and effective.

Worldwide, there has been a rush of guidelines for the evaluation of medical procedures with the more recent efforts acknowledging the differences between pharmaceuticals, surgical procedures and diagnostic tests. Unfortunately, the requirements of these guidelines are based more on what should be, rather than what is, since there would appear to have been less of a rush to generate the evidence required by these guidelines. As a consequence, the majority of negative recommendations from the MSAC continue to be based on insufficient evidence.

### Discussion and implications for health policy

#### Proposed timetable for the processing of applications to the MSAC

Presently there is no set timetable for the processing of applications to the MSAC. Despite the fact that one of the most crucial stages, the evaluation of the evidence, is given a set time of three months, unexplained delays in other stages of the process have resulted in applications taking in excess of two years to generate a new MBS Item Number. Table 9 shows a possible timetable for the processing of applications covering the major steps in the process. A set timetable would add a degree of certainty to the process for the Applicant and would eliminate the huge variation in the current processing time.

**Table 9:** Possible timetable for the application process

**Week**	**Process**
Week 0	Application lodged with the Health Technology Section.
Week 1	Written information to Applicant that application has been received and deemed eligible.
Week 2	Project Officer from the Health Technology Section allocated.
Weeks 2 – 8	Formation of Advisory Panel – letters sent out to relevant 'Craft Groups' for nominations.
Weeks 2 – 8	Evaluators appointed by Health Technology Section and requested to draft a protocol.
Week 10	First meeting of the Advisory Panel and Evaluators – refinement of draft protocol.
Week 12	Draft protocol sent out to Applicant for comments.
Week 14	Comments on draft protocol reviewed by Chairperson of the Advisory Panel and other members of panel if necessary.
Weeks 15 – 28	Evaluators evaluate Application.
Week 29	Evaluators draft Assessment Report presented to the Advisory Panel.
Weeks 30 – 31	Any reviews of draft Assessment Report carried out by the Evaluators.
Weeks 31 – 35	Reviewed draft Assessment Report sent out to Applicant for comments.
Week 36	Response to the Applicants comments to the Advisory Panel by Evaluators.
Next scheduled MSAC Meeting	Assessment Report (complete with recommendation by the Advisory Panel) and Applicant's comments presented to the MSAC meeting (held every three months).
2 weeks post Meeting.	Recommendation of the MSAC sent to the Minister.
6 weeks post Meeting.	Decision by Minister of Health and Ageing.

#### Appointment of Advisory Panels and the role of the Australian Medical Association (AMA)

The clinical and economic benefits of a positive MSAC recommendation can be dramatically reduced if the recommendation is not arrived at in a timely fashion. The MSAC process presently averages two years between the acceptance of the application and the listing of the new Item Numbers on the MBS. A major cause of this lengthy delay is the amount of time spent forming the Advisory Panel and agreeing on the dates of the first and subsequent meetings of this panel.

Currently, when a new application to the MSAC is received by the Medicare Benefits Branch, one of the first steps in the process is to write to the relevant medical association (Craft Group) and ask for nominations for positions on the Advisory Panel. This part of the process is open-ended in terms of timing of the response from the Craft Group and can take several months.

An example of the make up of expertise of the membership of an Advisory Panel is that for Application 1089, brachytherapy for the treatment of prostate cancer. This panel included a nuclear medicine specialist (Chair and MSAC member), two urologists, two radiation oncologists and, a consumer representative.

Discussions with the Australian Medical Association (AMA) and Craft Groups aimed at formulating guidelines, including timing, for the nomination process could facilitate a more rapid appointment of Advisory Panel members and allow the first Advisory Panel meeting to be convened within six weeks of the lodgement of an application.

#### Interim funding

Currently, where the evidence is inconclusive but suggests that the procedure could be at least as safe and possibly more effective and cost-effective as the existing comparable procedure, the MSAC may recommend interim funding for three years subject to the condition that additional data be collected to allow further and longer term evaluation of the procedure.

In its submission to the productivity commission inquiry into impact of advances in medical technology on health care expenditure in Australia the Faculty of Radiation Oncology (RANZCR) stated that "the implementation of new technology into Australia is affected by perceptions of efficacy, international experience, and MSAC approval for funding under the MBS. The introduction of new technology is not necessarily consistent with MSAC requirements in that the lag time to generate high-level evidence of benefit is often not consistent with clinical imperatives to introduce new technology. Furthermore, a chicken and egg situation can develop where funding for equipment is needed to generate evidence of effectiveness."[12]

RANZCR went on to say that 'the nature of equipment-based developments and the process of obtaining sufficient data for approval is a complex one, and there has been at least one instance in the Faculty's field (brachytherapy for prostate cancer) in which MSAC provided an interim approval. There have been challenges in collecting data to gain ongoing approval of MBS funding, especially when this is undertaken in an arm's-length manner from the equipment manufacturers, along the lines of clinical trial data collection protocols."

If interim funding in an expanded form, subject to the on-going collection of data, is to be a solution to the 'insufficient evidence' problem, there are clearly a number of issues that need to be resolved. Foremost of these issues are the funding of the cost of the data collection, the duration of the data collection and, who will be responsible for the collection of the data. A major drawback of an interim funding system is the problems associated with the discontinuation of funding should the data collected show the procedure not to be cost-effective. Owing to the fairly recent nature of the implementation of the MSAC process only one application that was initially granted interim funding has been reassessed. This application was brachytherapy for the treatment of prostate cancer (Application 1089) and it has been granted an extension of interim funding in order to allow more time for data collection.

A rare example of a government funded audit of a procedure granted interim funding by the MSAC is the audit of endoluminal repair of abdominal aortic aneurysms (Application 1006). The 1999 MSAC report on this procedure found that although it appeared to be effective in the short-term, there was insufficient evidence relating to the long-term safety and effectiveness. The Australian Audit of Endoluminal Graft (ELG) Repair of Abdominal Aortic Aneurysms (AAA) was established in 1999 to evaluate the mid- to long-term safety and efficacy of the procedure in the Australian setting. Results from the audit will help to inform funding decisions made by the Australian Government [14].

As at May 2005, this audit based on procedures carried out between November 1999 and May 2001 is on-going with a cost already measured in the hundreds of thousands of dollars.

Unlike pharmaceuticals, the financial beneficiary from the reimbursement of a new medical procedure can be difficult to identify. This raises the question of who should pay for the production, collection and analysis of the evidence. Table 4 shows that for the four year since July 2000, 86% of applications received by the MSAC have come from industry. This is due to the fact that these new medical procedures involve the use of new technology manufactured or distributed by medical device, medical equipment or diagnostic companies and an MBS listing is crucial to the commercial viability of these products.

Clearly, industry needs to be informed, and thus aware of the minimal level of evidence required by the MSAC process. If industry is to take a more active financial role in the generation of evidence it may also be justified in seeking a greater role in the process. At this stage industry has no representative on either the MSAC or any of the Advisory Panels. With new procedures that result from the development of substantial capital equipment rather than new variations of existing procedures (experimentation), for example robotically assisted minimally invasive surgery, the collection of affordable and acceptable clinical data should be possible.

#### Ongoing and large scale evaluation of the cost effectiveness and the incorporation of economic considerations into guidelines

In their 2001 paper, Eccles et al [15] explored the methods of incorporating cost issues within clinical guidelines and concluded that unlike other areas of guideline development, there is little practical or theoretical experience to direct the incorporation of cost issues within clinical guidelines.

In 2005 Richardson [16] concluded that:

"The scale of present evaluation activities is inadequate. In an industry absorbing 9 percent of the GDP – the country's largest industry – there should be ongoing and large scale evaluation and re-evaluation of the cost effectiveness of the services provided. Evaluation should be based upon a comparison with the full spectrum of substitute services. A failure to do this almost certainly ensures that there will be widespread and significant allocative inefficiency in the level and mix of services."

Despite the fact that the MSAC was the first of its kind in the world, the National Institute for Clinical Excellence (NICE) has overtaken it in terms of expertise due to a far greater allocation of resources. Presently the Health Technology Section that manages the evaluation process is understaffed and as a consequence struggling to cope with the workload. There is an urgent need for an injection of staff qualified to deal with not only the day to day administration of the evaluation process but also the development of methodology aimed at generating acceptable clinical evidence upon which to base cost-effectiveness evaluations. Methodology developed for these new procedures could also be applied to existing procedures that have raised some concern. A major policy issue that has become clear as a result of the evaluation of new procedures is that many of the existing 'gold standards' used as comparators are anything but gold and are based on little or no real evidence.

## Conclusion

New medical procedures are often the result of a process of experimentation rather than formally conducted research. The medical procedures being evaluated by the MSAC are, in the majority of cases, procedures that have been carried out elsewhere in the word for some time. Ironically this includes diagnostic tests using capital equipment developed in Australian itself. This raises the question as to exactly why there is such a low rate of positive recommendations from the MSAC. The answer may lie with what is considered to be an acceptable level of evidence and the fact that we have yet to develop a financially viable formal process for the generation of this level of evidence in Australia or elsewhere in the world.

The key characteristics of this clinical evidence are affordability, timeliness, systematic collection, unbiased and, not anecdotal.

When possible, interim funding of a new procedure can ensure the systematic collection of evidence while allowing the use of the new procedure. The answer to the timeliness question lies in a combination of a greater use of interim funding as well as the elimination of the unproductive time usage that exists in the current MSAC process.

Affordability and the question of who should pay for the generation, collection and analysis of the clinical evidence is perhaps the most difficult to answer especially in the case where the new procedure is the result of a process of experimentation with an old procedure. In cases where new technology is used, industry may be able to play a role here. However, this must be dependant on industry having a greater role in the MSAC decision making process.

Up to this point in time the use of economics and cost-effect analysis has not been feasible due to a lack of acceptable levels of clinical evidence. Currently, only the cost side of the equation is possible in the vast majority of new medical procedures with this often working against the new procedure since virtually all new procedures involve the use of expensive new technology.

Unless a cost-effective way is found to collect acceptable levels of evidence proving the clinical effectiveness of these new procedures, formal processes of evaluation such as that used by the Australian MSAC since 1998 will continue to run a very high risk of committing Type II errors, that is, denying access to medical procedures that are beneficial and efficient rather than risk a Type I error, approval of a non-beneficial procedure.

## Abbreviations

MSAC – Medical Services Advisory Committee

MBS – Medicare Benefits Schedule

PBAC – Pharmaceutical Benefits Advisory Committee

MBCC – Medicare Benefits Consultative Committee

PBS – Pharmaceutical Benefits Scheme

MBB – Medicare Benefits Branch

TGA – Therapeutic Good Administration

PBB – Pharmaceutical Benefits Branch

NHMRC – National Health and Medical Research Council

RCT – Randomised Controlled Trial

GDG – Guideline Development Group

RANZCR – Faculty of Radiation Oncology

ELG – Endoluminal Graft

AAA – Abdominal Aortic Aneurysms

## Competing interests

• The author declares that she has no competing interests.

• The author consults to industry in the capacity of a reimbursement specialist and currently has several applications being processed by the MSAC. This makes the author the single major contributor of MSAC applications. The author's first application was lodged in 1999 – Application 1029.

• The author is also currently enrolled as a DBA Candidate at the Macquarie Graduate School of Management (MGSM).

## Authors' contributions

Sole author – except where acknowledged, all analysis and conclusions in this paper are the author's.
